# Effects of Bike-Fitting on Lower Back Pain in Cyclists: A Systematic Review

**DOI:** 10.7759/cureus.101718

**Published:** 2026-01-17

**Authors:** Cristina Barrajón, Alejandro Legaz-Arrese, Rafel Cirer-Sastre, Isaac López-Laval, Sebastian Sitko

**Affiliations:** 1 Nursing, Hospital de San Juan de Alicante, Alicante, ESP; 2 Physiatry and Nursing, University of Zaragoza, Zaragoza, ESP; 3 National Institute of Physical Education of Catalonia (Institut Nacional d'Educació Física de Catalunya), Universitat de Lleida, Lleida, ESP

**Keywords:** bike-fitting, biomechanics, cycling, lower back pain, sports medicine

## Abstract

Lower back pain (LBP) is one of the most common overuse complaints among cyclists and is frequently linked to suboptimal bicycle configuration. Bike-fitting seeks to optimize the interaction between rider and bicycle to enhance comfort, efficiency, and injury prevention. This systematic review examined the effects of bike-fitting interventions on LBP in cyclists. A comprehensive search was conducted in PubMed, Scopus, Web of Science, SPORTDiscus, and the Cochrane Library up to October 2025 to identify studies evaluating any bike-fitting or ergonomic adjustment aimed at reducing LBP or related discomfort. Methodological quality was assessed using the Physiotherapy Evidence Database (PEDro) scale for randomized trials and the Methodological Index for Non-Randomized Studies (MINORS) checklist for non-randomized studies. Three studies met the inclusion criteria: one randomized controlled trial and two quasi-experimental designs. Interventions spanned static and dynamic fitting procedures, as well as ergonomic saddle adjustments, with follow-up periods ranging from 30 days to six months. All studies reported significant reductions in LBP or discomfort following individualized fitting. These improvements were primarily associated with optimized pelvic tilt, improved spinal alignment, and more efficient lower-limb kinematics. Although methodological quality was generally moderate to high, variability in intervention protocols and relatively short follow-up durations limit the generalizability of the findings. Overall, bike-fitting, particularly when personalized, dynamic, and tailored to workload demands, appears to be an effective strategy for reducing LBP and enhancing comfort in cyclists. However, further high-quality research is needed to standardize fitting parameters, incorporate objective biomechanical assessments, and determine the long-term efficacy of these interventions.

## Introduction and background

Lower back pain (LBP) is defined as pain, muscle tension, or stiffness localized below the ribs and above the gluteal folds, with or without leg pain (sciatica). The World Health Organization (WHO) estimated in 2020 that LBP affected more than 619 million people worldwide, making it one of the most prevalent and disabling musculoskeletal disorders globally [1]. Its burden extends far beyond clinical symptoms, contributing substantially to reduced physical function, decreased productivity, and diminished quality of life. Among physically active individuals, the presence of LBP can also limit training consistency and performance outcomes, underscoring the importance of preventive strategies in both general and athletic populations.

Within endurance sports, cycling represents a unique context in which repetitive motion and sustained postures may predispose athletes to LBP. According to the literature, LBP is the most common musculoskeletal complaint among cyclists, affecting at least one-third of this population [2-4]. The pain typically develops gradually and is associated with the prolonged forward-flexed posture required to maintain aerodynamic efficiency. However, the mechanisms by which repetitive stress, vibration, and sustained flexion contribute to lumbar discomfort remain incompletely understood. It also remains unclear how the interaction between intrinsic factors, such as flexibility, core stability, and prior injury, and extrinsic factors, such as bicycle geometry or training load, modulates the risk of developing LBP.

Biomechanical studies have provided valuable insights into these mechanisms, revealing that deviations in posture, pedaling asymmetry, and excessive lumbar flexion can increase mechanical stress on the spine [5]. Through the assessment of kinematics, kinetics, and electromyographic activity, this research has shown that the cyclist’s position on the bicycle directly affects spinal loading, pelvic rotation, and muscle activation patterns. These findings have not only improved understanding of the pathomechanics of cycling-related pain but also contributed to developing interventions that aim to reduce musculoskeletal strain and improve performance [6].

Within this biomechanical framework, professional bike-fitting has emerged as a fundamental preventive and corrective tool. Improper adjustment of saddle height, handlebar position, or frame geometry can alter spinal curvature, increase shear forces, and overload paraspinal musculature, particularly during long rides [5]. Conversely, precise adjustment of these parameters, taking into account individual characteristics such as flexibility, anthropometrics, riding discipline, and competitive goals, can reduce mechanical stress on the lumbar spine and enhance comfort [6]. Classical fitting approaches have traditionally relied on static anthropometric measurements and predefined joint angle ranges, whereas modern fitting integrates dynamic assessments during pedaling, incorporating kinematic, kinetic, and pressure-related parameters. Advances in technology have also transformed fitting practices: traditional static measurements are increasingly being replaced or complemented by dynamic motion capture systems, pressure mapping, and three-dimensional analyses that allow real-time assessment of joint angles, pelvic motion, and load distribution.

Despite these technological innovations and the widespread application of professional fitting services, the scientific evidence supporting their effectiveness in preventing or alleviating LBP remains limited and heterogeneous. Most studies differ in methodology, outcome measures, and the parameters evaluated, making it difficult to draw firm conclusions or develop standardized clinical guidelines. Furthermore, the relative efficacy of classical (static) versus modern (dynamic) fitting methods is not well established, and few studies have examined how these interventions affect long-term pain outcomes, posture, or performance.

Given these considerations, the objectives of the present study were twofold: (1) to systematically gather and analyze the existing literature on the effectiveness of bicycle fitting interventions for the reduction of LBP in cyclists, and (2) to evaluate potential differences in efficacy between classical and modern fitting approaches. By synthesizing current evidence, this review seeks to clarify the role of professional fitting in managing cycling-related LBP and to identify priorities for future research aimed at developing standardized, evidence-based fitting protocols.

## Review

Materials and methods

Search Strategy

This systematic review was conducted following the Preferred Reporting Items for Systematic Reviews and Meta-Analyses (PRISMA) guidelines and was retrospectively registered in the International Prospective Register of Systematic Reviews (PROSPERO) database (ID CRD420251184737). A comprehensive literature search was performed across the databases PubMed, Scopus, Web of Science, SPORTDiscus, and Cochrane Library, from their inception to October 2025. The search combined terms related to cycling, bike-fitting, and LBP using Boolean operators: (“cycling” OR “cyclist”) AND (“bike fit” OR “bike fitting” OR “bicycle fitting” OR “bicycle adjustment” OR “bike position”) AND (“low back pain” OR “back pain” OR “lumbar pain”). Reference lists of relevant articles were manually screened to identify additional eligible studies. Full electronic search strategies for all databases have been provided in Appendix A.

Eligibility Criteria

Studies were included if they involved recreational or competitive cyclists of any age or sex who underwent any form of bike-fitting or bicycle adjustment procedure, such as modifications in saddle height or setback, handlebar position, or comprehensive static or dynamic fitting protocols. Eligible studies were required to include a comparison condition (e.g., pre- versus post fitting, usual position, or alternative fitting approach) and to report outcomes related to LBP incidence, severity, or associated biomechanical and postural parameters. Randomized controlled trials, quasi-experimental designs, cohort, and case-control studies were considered. Studies were excluded if they involved non-cyclist populations, interventions unrelated to bike-fitting (such as strength training or physiotherapy programs), or if they were reviews, conference abstracts, case reports, or not published in English or Spanish.

Study Selection and Data Extraction

**Figure 1:**
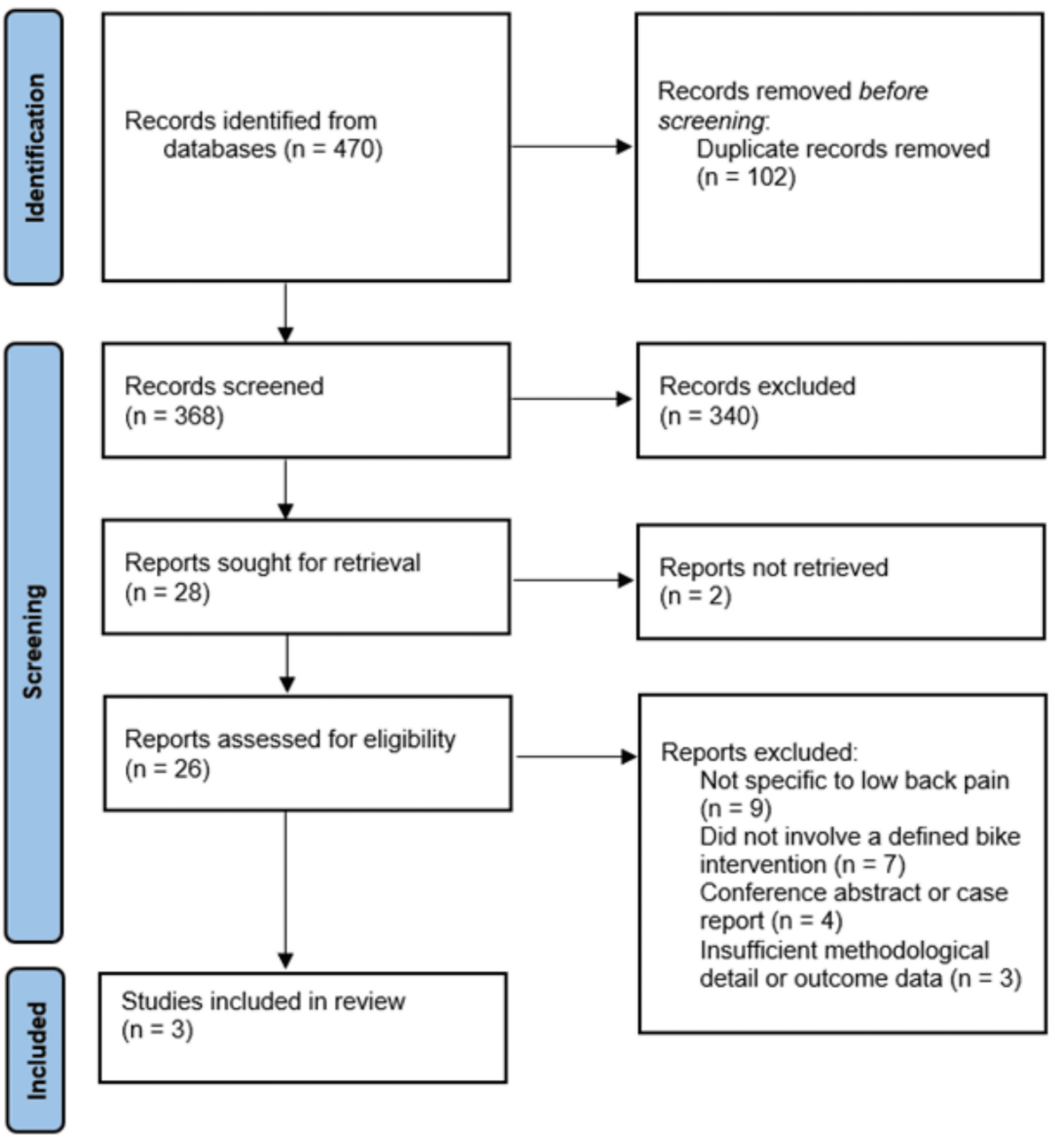
A PRISMA flowchart outlining the study selection process PRISMA: Preferred Reporting Items for Systematic Reviews and Meta-Analyses

Data extraction was performed using a predefined extraction template agreed upon by the reviewers. Primary outcomes were measures related to LBP, including incidence, prevalence, intensity, or changes in pain symptoms following bike fitting interventions. Secondary outcomes included biomechanical, postural, and kinematic variables (e.g., spinal posture, joint angles, pelvic motion), pressure distribution, comfort-related measures, and performance-related variables when reported. Two independent reviewers screened titles and abstracts for relevance. Full texts of potentially eligible articles were then assessed against the inclusion criteria. Disagreements were resolved through discussion or consultation with a third reviewer. Extracted data included study characteristics (authors, year, country, design, sample size), participant demographics, bike-fitting intervention details, outcomes assessed, and main results. When key data were missing or unclear, corresponding authors were contacted via email. If no response was received, data were extracted as reported, and missing values were indicated as ‘not available.’ Figure 1 provides a graphic summary of the study selection process.

Quality Assessment

Each study was independently evaluated by two reviewers, and discrepancies were resolved by consensus. The overall level of evidence and risk of bias were summarized narratively. Given the heterogeneity in study design (randomized and non-randomized), methodological quality was assessed using design-appropriate tools: the Physiotherapy Evidence Database (PEDro) scale for randomized controlled trials and the Methodological Index for Non-Randomized Studies (MINORS) checklist for quasi-experimental studies [7,8]. The randomized controlled trial by Dias Scoz et al. (2022) demonstrated high methodological quality (PEDro score = 8/10) [9], showing adequate randomization, allocation concealment, and assessor blinding. The quasi-experimental and pre-post studies (Scoz et al., 2021; Salai et al., 1999) scored 12/16 and 11/16 on the MINORS scale, respectively, reflecting moderate methodological quality with clear aims, appropriate endpoints, and adequate follow-up, but lacking blinding and control groups [10,11]. Table 1 represents a summary of the methodological quality assessment.

**Table 1:** Methodological quality assessment of the three included studies using the PEDro and MINORS scales PEDro: Physiotherapy Evidence Database; MINORS: Methodological Index for Non-Randomized Studies; N/A: not available

Criterion	Dias Scoz et al., 2022 (PEDro) [9]	Scoz et al., 2021 (MINORS) [10]	Salai et al., 1999 (MINORS) [11]
1. Clearly stated aim	Yes	Yes	Yes
2. Eligibility criteria specified	Yes	Yes	Yes
3. Random allocation	Yes	No	No
4. Concealed allocation	Yes	No	No
5. Groups similar at baseline	Yes	Yes	Yes
6. Blinding of participants	No	No	No
7. Blinding of therapists	No	No	No
8. Blinding of assessors	Yes	No	No
9. Adequate follow-up (>85%)	Yes	Yes	Yes
10. Intention-to-treat analysis	Yes	No	No
11. Between-group comparisons	Yes	N/A	Yes
12. Point estimates and variability data	Yes	Yes	Yes
13. Prospective data collection (MINORS)	N/A	Yes	Yes
14. Endpoints appropriate to aim (MINORS)	N/A	Yes	Yes
15. Unbiased assessment of endpoints (MINORS)	N/A	No	No
16. Follow-up period appropriate (MINORS)	N/A	Yes	Yes
17. Loss to follow-up <5% (MINORS)	N/A	Yes	Yes
18. Statistical analysis adequate (MINORS)	Yes	Yes	Yes
Total score (PEDro 0–10 / MINORS 0–16)	8	12	11

Data Synthesis

Given the expected heterogeneity in interventions, outcome measures, and study designs, a qualitative synthesis was prioritized. Results were grouped according to the type of bike fitting intervention and the outcome domain (pain reduction, posture, comfort, or performance). Where applicable, pain outcomes were expressed as mean differences or percentage changes from baseline, while comfort and fatigue scales were analyzed descriptively. Heterogeneity across studies was not quantitatively assessed, and no sensitivity analyses were performed due to the small number of included studies and methodological diversity. Potential reporting bias was not formally assessed because of the limited number of studies and the absence of a meta-analysis. No meta-analysis was conducted due to heterogeneity of study designs and outcome measures.

Results

Three studies met the inclusion criteria and were included in the qualitative synthesis. All examined the influence of bike-fitting or ergonomic adjustments on LBP and related musculoskeletal discomfort in cyclists.

Despite differences in design and methodology, the findings were consistent: all interventions resulted in measurable reductions in discomfort and pain following adjustments to the bicycle setup. The two studies employing 3D dynamic fitting systems reported significant short-term improvements in pain, comfort, and fatigue scores after 30 days, with moderate-to-large effect sizes. Similarly, the study that implemented a static ergonomic modification (anterior saddle tilt) demonstrated substantial long-term reductions in LBP over a six-month period.

Across studies, perceived discomfort and fatigue decreased in multiple body regions, including the back, neck, and knees, suggesting that optimized posture and alignment achieved through bike fitting may reduce localized mechanical stress and enhance overall cycling comfort. Kinematic data from the randomized trial further supported this interpretation, showing improvements in spinal alignment and movement symmetry. Table 2 shows the main characteristics of the studies included in the systematic review.

**Table 2:** Main characteristics of the studies included in the systematic review F: female; M: male; LBP: lower back pain; VAS: Visual Analog Scale; OMNI: OMNI Rating of Perceived Exertion

Study	Sample	Intervention	Outcomes Measured	Main Results
Salai et al., 1999 [11]	80 recreational cyclists (30F/50M), aged 17–72 years	Saddle tilt adjustment (10°–15° anterior inclination) based on fluoroscopic biomechanical analysis; follow-up duration: six months	LBP incidence and intensity (self-reported before and after adjustment)	After six months, 72% reported complete resolution of LBP, 20% major improvement, and 7% no change (p < 0.01).
Scoz et al., 2021 [10]	160 amateur mountain bikers (120M/40F), mean age 38.7 ± 8.0 years	Standardized 3D kinematic bike fitting using the Retül system; adjustments made to ensure recommended joint ranges; follow-up duration: 30 days	Pain (VAS), comfort (FEEL), and fatigue (OMNI) scales before and 30 days after fitting	Significant improvements in all measures (p < 0.001); Moderate improvements for LBP.
Dias Scoz et al., 2022 [9]	72 trained cyclists (49M/23F), mean age 36.5 ± 7.9 years	Randomized controlled trial comparing 3D dynamic bike fitting (Retül system) vs. usual setup; follow-up duration: 30 days	VAS discomfort, FEEL comfort, and kinematic parameters (joint angles, spinal posture) pre– and post intervention	The fitted group showed significant reductions in back discomfort (p < 0.001). Kinematic analysis showed reduced spinal flexion and improved lower-limb symmetry.

Discussion

This systematic review synthesized the available evidence on the effects of bike-fitting interventions on LBP in cyclists. Although only a limited number of studies met the inclusion criteria, the findings consistently indicate that individualized bicycle adjustments can alleviate pain and discomfort, improve posture, and enhance overall cycling comfort. These results are consistent with the growing body of literature suggesting that biomechanical optimization of the cyclist-bicycle interface contributes to both performance improvement and injury prevention [12].

Cycling practice induces specific adaptations in spinal morphology compared with non-cyclists, including increased pelvic tilt, greater lumbar flexion capacity during trunk flexion, and enhanced thoracic kyphosis in the standing position [13]. Moreover, cyclists experiencing LBP often display altered positions on the bicycle, underscoring the importance of assessing the cyclist-bicycle interaction. However, previous research indicates that it is not always possible to determine whether these positional changes are a cause or a consequence of LBP [14].

Across the included studies, both static and dynamic bike-fitting approaches demonstrated meaningful reductions in perceived pain and improvements in comfort and posture. The randomized controlled trial by Dias Scoz et al. (2022) provided the most robust evidence, reporting significant reductions in pain and improved postural symmetry compared with a control group [9]. Similarly, the quasi-experimental studies by Salai et al. (1999) and Scoz et al. (2021) observed decreased discomfort and enhanced spinal alignment following ergonomic interventions [10,11]. Collectively, these findings suggest that optimizing bicycle geometry can mitigate the mechanical and neuromuscular stresses associated with prolonged cycling postures, thereby reinforcing the role of professional fitting in the management and prevention of cycling-related LBP.

The underlying mechanisms of cycling-related LBP appear to be multifactorial. Suboptimal positioning, such as excessive saddle setback, inappropriate saddle height, or a pronounced handlebar drop, can increase lumbar flexion, pelvic rotation, and shear forces on the spine, thereby contributing to LBP [15,16]. Conversely, individualized fitting can alleviate these stresses by optimizing biomechanical alignment, redistributing load between the pelvis and upper limbs, and reducing compensatory muscle activation. Swart and Holliday (2019) emphasized that dynamic assessments more accurately capture these biomechanical variations, as joint angles and pelvic tilt change substantially with increasing intensity and fatigue. Indeed, kinematic analyses indicate that greater cycling workloads are associated with increased hip extension and thoracic flexion, which can alter spinal posture and potentially predispose cyclists to overuse symptoms if not properly adjusted. The use of three-dimensional motion analysis and saddle pressure mapping offers new opportunities to detect and correct maladaptive postures, providing a more evidence-based alternative to traditional static fitting approaches [12]. Based on this evidence, future bike-fitting studies should incorporate assessments performed under fatigue conditions to better reflect real-world demands. Epidemiological evidence further highlights the importance of appropriate bike-fitting and training management. Cross-sectional studies involving more than 1000 cyclists have reported a lifetime LBP prevalence exceeding 50%, with professional riders exhibiting even higher rates, likely due to greater training volumes and aerodynamic demands [4,17,18]. Among recreational cyclists, inadequate bicycle fit, prolonged lumbar flexion, and excessive weekly mileage (>160 km) have been identified as major contributors to LBP [19]. Additional risk factors include improper saddle tilt, low stem height, and insufficient core stability [13]. Conversely, supervised training and technique-oriented interventions, such as pedaling efficiency coaching, have been negatively associated with LBP prevalence [4]. Research consistently supports individualized bike-fitting, particularly regarding saddle height and handlebar position, to optimize comfort and reduce pain. Dynamic fitting approaches that consider individual flexibility and training history are especially recommended. Although a recent consensus statement outlined standardized methods for measuring bicycle setup dimensions and best practices for collecting kinematic data, the evidence supporting optimal values for many fitting parameters remains limited [20]. Saddle height appears to be the most extensively studied variable, with previous research recommending a knee flexion angle of approximately 25°-35° under static conditions and 30°-40° during dynamic assessment [13]. Additionally, a slight anterior saddle tilt (10°-15°) may help reduce LBP. However, there is still a lack of clear evidence-based guidance for other parameters, such as crank length, handlebar height, Q-factor, and cleat position [21]. Dynamic assessments may be particularly valuable, as they capture workload-dependent kinematic adaptations often overlooked in static fits. For example, cyclists tend to increase ankle dorsiflexion and hip extension with rising intensity, which can alter lumbar curvature and load transmission through the spine. Consequently, performing dynamic fittings at realistic training intensities is likely to yield more relevant and sustainable ergonomic benefits. Nevertheless, several methodological limitations temper the strength of the current conclusions. The available evidence remains limited, and most studies rely primarily on subjective measures of pain and comfort, with minimal objective biomechanical or physiological validation. The limited number of available studies and the lack of registered protocols may introduce reporting bias. Additionally, the certainty of the evidence remains moderate to low due to methodological variability. Furthermore, participant blinding is generally infeasible, and follow-up periods are typically short, making it difficult to assess the long-term persistence of improvements. Future research should aim to establish standardized fitting protocols, validated kinematic reference values for the hip and spine, and longitudinal randomized trials to better elucidate the causal relationship between bike-fitting and pain outcomes. In addition, individual factors such as flexibility, prior injury history, and training load should be systematically considered, as they likely interact with fitting parameters and influence pain response [13]. Finally, it should also be noted that differences in cycling discipline, equipment, and training environment may influence both the prevalence of LBP and the effectiveness of fitting interventions, highlighting the need for discipline-specific analyses. From a practical perspective, these findings underscore the value of professional bike-fitting as part of a comprehensive management strategy for cyclists with, or at risk of, LBP. This approach should be complemented by flexibility and core-stability training, gradual load progression, and attention to posture maintenance under fatigue. While the short-term benefits of ergonomic fitting appear well supported, future research should investigate whether such interventions also promote long-term performance improvements and reduce the recurrence of cycling-related injuries. In addition, advances in wearable sensors and artificial intelligence may soon allow real-time optimization of bicycle fit, offering new opportunities for individualized injury prevention strategies. In summary, individualized bike-fitting, particularly when performed dynamically, appears to substantially reduce LBP and improve comfort in cyclists. These benefits are likely mediated by improved biomechanical alignment, reduced spinal loading, and optimized muscle coordination. These findings have direct implications for both clinicians and coaches, emphasizing the need to integrate professional bike-fitting into rehabilitation and performance optimization programs for cyclists of all levels. However, high-quality, standardized research is still needed to establish evidence-based guidelines for fitting practice and to confirm the effectiveness of dynamic fitting as both a therapeutic and preventive strategy for cycling-related LBP.

## Conclusions

Individualized bike-fitting, especially when performed dynamically and at training-specific intensities, appears effective in reducing LBP and discomfort in cyclists. Adjustments that improve pelvic orientation and spinal alignment are likely to decrease mechanical stress and enhance comfort. However, evidence remains limited by methodological heterogeneity and short follow-ups. Future research should standardize dynamic fitting protocols, incorporate objective biomechanical assessments, and evaluate long-term outcomes. Bike-fitting should be considered a key component of comprehensive strategies for cycling injury prevention and comfort optimization.

## Appendices

Appendix A

Full Electronic Search Strategies

Databases and Search Period Electronic searches were conducted in the following databases during October 2025: - PubMed (MEDLINE) - Scopus - Web of Science Core Collection - SPORTDiscus - Cochrane Library No restrictions regarding publication year were applied. Searches were limited to studies published in English or Spanish.

PubMed (MEDLINE)

("cycling"[Title/Abstract] OR "cyclist"[Title/Abstract]) AND ("bike fit"[Title/Abstract] OR "bike fitting"[Title/Abstract] OR "bicycle fitting"[Title/Abstract] OR "bicycle adjustment"[Title/Abstract] OR "bike position"[Title/Abstract]) AND ("low back pain"[Title/Abstract] OR "back pain"[Title/Abstract] OR "lumbar pain"[Title/Abstract]) Filters applied: Humans

Scopus

TITLE-ABS-KEY ( cycling OR cyclist ) AND TITLE-ABS-KEY ( "bike fit" OR "bike fitting" OR "bicycle fitting" OR "bicycle adjustment" OR "bike position" ) AND TITLE-ABS-KEY ( "low back pain" OR "back pain" OR "lumbar pain" )

Web of Science Core Collection

TS=(cycling OR cyclist) AND TS=("bike fit" OR "bike fitting" OR "bicycle fitting" OR "bicycle adjustment" OR "bike position") AND TS=("low back pain" OR "back pain" OR "lumbar pain") Indexes searched: SCI-EXPANDED, SSCI

SPORTDiscus (EBSCOhost)

AB ( cycling OR cyclist ) AND AB ( "bike fit" OR "bike fitting" OR "bicycle fitting" OR "bicycle adjustment" OR "bike position" ) AND AB ( "low back pain" OR "back pain" OR "lumbar pain" )

Cochrane Library

(cycling OR cyclist) AND ("bike fit" OR "bike fitting" OR "bicycle fitting" OR "bicycle adjustment" OR "bike position") AND ("low back pain" OR "back pain" OR "lumbar pain") Searches were conducted in the Cochrane Database of Systematic Reviews and CENTRAL.

Additional Search Methods

The reference lists of all included studies and relevant reviews were manually screened to identify additional eligible articles not captured through database searches.
